# Extreme Metal Music and Anger Processing

**DOI:** 10.3389/fnhum.2015.00272

**Published:** 2015-05-21

**Authors:** Leah Sharman, Genevieve A. Dingle

**Affiliations:** ^1^School of Psychology, University of Queensland, Brisbane, QLD, Australia; ^2^Centre for Youth Substance Abuse Research, University of Queensland, Birsbane, QLD, Australia

**Keywords:** metal music, anger, emotion processing, heart rate, arousal

## Abstract

The claim that listening to extreme music causes anger, and expressions of anger such as aggression and delinquency have yet to be substantiated using controlled experimental methods. In this study, 39 extreme music listeners aged 18–34 years were subjected to an anger induction, followed by random assignment to 10 min of listening to extreme music from their own playlist, or 10 min silence (control). Measures of emotion included heart rate and subjective ratings on the Positive and Negative Affect Scale (PANAS). Results showed that ratings of PANAS hostility, irritability, and stress increased during the anger induction, and decreased after the music or silence. Heart rate increased during the anger induction and was sustained (not increased) in the music condition, and decreased in the silence condition. PANAS active and inspired ratings increased during music listening, an effect that was not seen in controls. The findings indicate that extreme music did not make angry participants angrier; rather, it appeared to match their physiological arousal and result in an increase in positive emotions. Listening to extreme music may represent a healthy way of processing anger for these listeners.

## Introduction

Music is a widely available form of media with the ability to influence attitudes and manipulate emotions (Juslin and Sloboda, 2010; Wheeler et al., 2011), and listeners are drawn to music that reflects or improves their emotional state (Saarikallio, 2011; Thoma et al., 2012; Papinczak et al., 2015). Heavy metal, emotional (emo), hardcore, punk, screamo, and each of their subgenres form the category of “extreme” music. Extreme music is characterized by chaotic, loud, heavy, and powerful sounds, with emotional vocals, often containing lyrical themes of anxiety, depression, social isolation, and loneliness (Shafron and Karno, 2013). Perhaps, due to these musical characteristics, it has been claimed that extreme music leads to anger, and expressions of anger such as aggression, delinquency, drug use, and suicidal acts (Selfhout et al., 2008). Certainly, evidence is available regarding the effect of a listeners’ emotional state on their choice and preference for music listening even when angry. Research on anger processing has found that approach motivation (defined as the impulse to move forward) may be activated by anger (Carver and Harmon-Jones, 2009), such that after experiencing anger we then look to act out approach motivated behaviors, for example, angry facial expression and physical retaliation. Considering the highly arousing nature of the music, along with negative themes commonly contained in the lyrics, extreme music has been interpreted as *eliciting* anger among its listeners, and that this may activate aggressive behaviors (Gowensmith and Bloom, 1997). It is equally plausible, however, that extreme music may be chosen when a listener is angry, because the arousing nature of the music may match the already present internal arousal of the listener and allow him/her to explore and process this emotional state. This study will explore these alternative hypotheses about the influence of extreme music listening on anger processing in a sample of extreme music listeners under controlled experimental conditions.

### Extreme music

Extreme music genres began to emerge in the early 1970s with the decline of the “free love” and optimistic culture of the 1960s (Stack et al., 1994). Due to the consequences of the 1960s era of drug experimentation, decline of peaceful protest movements, and the continuation of the Vietnam War, angry and pessimistic themes began to emerge in new genres of music (Reddick and Beresin, 2002). Thus, punk and heavy metal music were dedicated to notions of anarchy and destruction (Stack et al., 1994; Reddick and Beresin, 2002; Lozon and Bensimon, 2014). Following the rise of punk and heavy metal, a range of new genres and subgenres surfaced. Hardcore, death metal, emotional/emotional-hardcore (emo), and screamo appeared throughout the 1980s, gradually becoming more a part of mainstream culture. Each of these genres and their subgenres are socio-politically charged and, as mentioned earlier, are characterized by heavy and powerful sounds with expressive vocals.

At the forefront of controversy surrounding extreme music is the prominence of aggressive lyrics and titles, such as “Pure Hatred” by Chimaira and “Violent Revolution” from the band Kreator. In a series of five experiments involving first year psychology students and student volunteers (unselected in terms of demographic characteristics or musical preference), Anderson et al. (2003) played musically equivalent songs with and without violent lyrics to the participants. They found that listening to songs with violent lyrics increased participants’ state hostility relative to listening to non-violent songs. However, this effect was fleeting and it was disrupted when the participants did intervening tasks. Other research shows that lyrical content is one of the mechanisms linking music with emotional response, although many other musical variables, contextual variables, and individual listener variables also play a role (Juslin and Västfjäll, 2008; Juslin et al., 2008).

The powerful vocals that exist in the most extreme genres such as screamo, where nearly all lyrics are screamed at the listener, may account for the perception by outsiders that this music is angry. From this stems a stereotype that extreme music fans, and especially heavy metal fans, are more aggressive, agitated, and more aroused than the general public (Arnett, 1991; Alessi et al., 1992). Furthermore, extreme music has been held responsible for social problems like depression, suicide, aggressive behavior, and substance misuse (Shafron and Karno, 2013). Some researchers have used the term “problem music” in reference to these genres, meaning music that is associated with psychological vulnerability and social deviance (North and Hargreaves, 2006; Bodner and Bensimon, 2014; Lozon and Bensimon, 2014). In the case of substance use, for example, a correlational study of 7,324 Dutch adolescents found that when all other factors were controlled, preferences for punk/hardcore, techno/hardhouse, and reggae music were associated with more substance use, whereas preferences for pop and classical music were linked to less substance use. A preference for rap/hip-hop only indicated elevated smoking among girls and, interestingly, a preference for heavy metal was associated with *less* smoking among boys and *less* drinking among girls (Mulder et al., 2009). This evidence does not support a causal view. Extreme music typically does not contain themes of illicit drug use, although some songs do contain lyrics related to alcohol use. Indeed, the movement known as “straight edge” is a subgenre of hardcore punk, whose adherents refrain from using alcohol, tobacco, and other recreational drugs. Furthermore, there are documented examples of rap music being used in therapeutic ways with samples of people who misuse substances (Baker et al., 2012; Lightstone, 2012).

A review by Baker and Bor (2008) found a relationship between various genres of music and antisocial behaviors, vulnerability to suicide, and drug use among young people. However, there was no evidence in these studies for a causal link, and it was instead suggested that music preference is a reflection of emotional vulnerability in these young listeners. More recently, Bodner and Bensimon (2014) investigated personality traits and uses of music to influence emotions among 548 middle class university students aged 18–43 years, who were subdivided into two groups based on their preference for “problem music” genres (*N* = 255 fans of heavy metal, punk, alternative rock, hip-hop, and rap) or “non-problem music” (*N* = 293 who did not endorse any of these in their top three musical genres). There were no differences between the two samples across the big five personality dimensions (extraversion, neuroticism, openness to experience, conscientiousness, and agreeableness). In terms of uses of music to influence emotions, there were no differences between groups in their use of music for entertainment and strong sensation; however, there were small differences in use of music for revival, diversion, emotional discharge, mental work, and solace. In each case, the problem music fans used music for emotion regulation slightly more than the non-problem music fans. The authors interpreted their findings to mean that listening to these types of music allows problem music fans to regulate their mood in a more sublimated way, instead of externalizing negative emotions, which in turn could lead to engaging in antisocial acts.

### Extreme music and anger

Some evidence is available regarding the effect of listeners’ emotional states on their choice and preference for music listening when angry. Shafron and Karno (2013) examined music preferences in a sample of 551 university students and divided the sample into two groups: those who preferred heavy metal and hard rock genres (57%) and the rest. The heavy music fans showed significantly higher symptoms of depression and anxiety than the non-fans; however, there was no difference between the two groups on trait anger. Gowensmith and Bloom (1997) found that heavy metal fans did not show an increase in anger after listening to heavy metal music. In this study, heavy metal music was highly arousing to both fans and non-fans, and in fact, measured state-arousal was greater among heavy metal listeners. Despite the arousing influence of the music, heavy metal fans displayed no difference in self-reported anger whether they were listening to a non-preferred music genre (country) or heavy metal. Non-fans, on the other hand, did display greater self-reported anger after listening to heavy metal. It is unclear whether the non-fans were angry as a result of the musical characteristics, or because they were being asked to listen to something they did not enjoy. So, although there is evidence that heavy metal increases state arousal (Stack et al., 1994; Gowensmith and Bloom, 1997), there is as yet insufficent evidence that it causes increased anger.

In a more naturalistic study, Labbé et al. (2007) found that after experiencing a state of induced stress or anger, participants listening to classical music chosen by the experimenter or their own self-selected “calming” music (of any genre) showed significant reductions in anger and anxiety. These reductions were evident in both self-reported ratings and in reduced physiological arousal (heart rate, respiration, and skin conductance) during music listening. In contrast, participants who listened to heavy metal after the stress induction did not reduce self-reported negative emotional states or physiological arousal. However, it is important to note that heavy metal was not a preferred music genre for these participants. This finding highlights the importance of personally selected music in determining the emotional response. Although this research suggests that a song considered relaxing by the listener should reduce anger and stress in the presence of a stressor, it remains to be seen whether this effect generalizes to extreme music genres.

### Considering the case of music and sadness

Related research on another negatively valenced emotion, sadness, might help to shed some light on music and anger processing. Some studies show that people listen to sad music when they are sad in order to improve their mood (Saarikallio and Erkkila, 2007). For instance, Papinczak et al. (2015) showed in both qualitative and quantitative studies with participants aged 15–25 years that they used music to immerse in negative moods such as sadness – a strategy that helped to process their sadness and to feel better. Similarly, a study of 65 adults from five countries found that when they were feeling sad, sad music helped these individuals to connect with their emotions through the music to fully experience sadness and consequently improve their affect (Van den Tol and Edwards, 2013). Despite evoking sadness, Finnish university students reported that they enjoy listening to sad music, and this effect was partly explained by personality traits such as openness to experience and empathy (Vuoskoski et al., 2012). On the other hand, some studies have reported that listening to sad music results in a more depressed mood among participants (Chen et al., 2007; Dillman Carpentier et al., 2008; Garrido and Schubert, 2015) – an effect that may be related to participants’ use of maladaptive emotion regulation strategies such as rumination. So, the influence of negatively valenced music on listeners appears to depend on the listening context, their current mood, and moderation by other personality traits.

### Study aims and hypotheses

To summarize the literature reviewed here, research on music and emotion supports the function of music to convey and elicit strong emotion. However, to date there has been a limited amount of research on extreme music genres and anger, with the exception of correlational studies showing an association, and one series of experiments claiming that listening to extreme music increases state hostility (Anderson et al., 2003). Thus, the current study sought to explore this question by recruiting extreme music listeners for an experimental study on the effects of extreme music listening (compared to a no music control condition) on anger processing. Given that personally selected music is capable of determining emotional responses (Labbé et al., 2007), participants were asked to bring along their personal music players to the experiment. In contrast to Labbé and colleagues’ study in which the participants were instructed to bring along music that they found relaxing, in the current study participants were allowed to listen to any music from their personal listening device that they preferred at the time.

Anger was operationalized in this study in terms of both subjective ratings of hostility and irritability and physiological recording of heart rate, which were expected to increase when participants experienced an increase in anger. The cardiovascular system is complex and has multiple regulatory subsystems from central and peripheral autonomic nervous systems and humoral influences (Bernison et al., 2007). Resting heart rate may be influenced by an individual’s age, aerobic fitness, posture, and activity levels. This is less of a concern with within-subjects designs such as was used in the current study, where the participant related factors are kept constant while the experimental factor (e.g., music listening or silence) is varied. Nevertheless, an increase in heart rate may reflect various psychological states including anger, stress, excitement, or fear. Heart rate should therefore be interpreted in combination with participants’ subjective ratings (of these psychological states) for a more accurate assessment of emotional response (Bernison et al., 2007).

According to the “problem music causes anger” line of reasoning, extreme music listeners who are angry would be expected to experience an *increase in anger* during music listening (as shown in an increase in heart rate during music listening and an increase in subjective anger ratings immediately following music listening). Thus, the first two hypotheses for investigation are:

Hypothesis 1a: that on a self-report measure of music and emotions, participants will endorse the statement that they listen to extreme music to fully experience their anger but will *disagree* with the statement that they listen to music to calm themselves down when feeling angry; and
Hypothesis 1b: that the participants’ subjective ratings and physiological measure (i.e., heart rate) of anger will increase during the anger induction and *will continue to increase* during music listening, and relative to participants in the no music (control) condition.

Another body of research indicates that listeners are drawn to music that is concordant with their current emotional state, and are able to use music as an emotion regulation technique (Saarikallio, 2011; Thoma et al., 2012; Papinczak et al., 2015). According to this “music regulates anger” line of reasoning, angered extreme music fans would be expected to listen to music that matches their anger and helps them to process it and feel better. Further, in Lozon and Bensimon (2014) review on problem music, they also concluded that listeners of music containing themes of aggression and suicidal ideation seemed to feel alleviated of angst and aggression after listening. Thus the alternative hypotheses are:

H2a: that on a self-report measure of music and emotions, participants will *agree* with the statements that they listen to music to fully experience anger, and that listening to music helps them to calm down when they are angry;
H2b: that the participants’ subjective ratings and physiological measure (i.e., heart rate) of anger will increase during the anger induction but *will not continue to increase* during music listening, and relative to participants in the no music (control) condition.
H2c: that, in accordance with the idea that extreme music may be a method for processing anger, participants in the music listening condition will feel better after music listening compared to the no music control participants, as shown by their endorsement of positively valenced emotions such as “relaxed” and “inspired.”

A secondary aim for the study was to analyze what the participants in the music condition selected from their own playlists to listen to when they were angry. This analysis will investigate the features of their chosen music in terms of genre, whether the songs contained angry lyrics, and the speed of tempo (beats/min).

H3: it was predicted that angry participants would select extreme music from their playlists that matched their anger in terms of high tempo and angry lyrics.

## Materials and Methods

### Participants

There were 40 people recruited to the study; however, one person’s data were unusable so the final sample consisted of 39 participants (72% male), with ages ranging from 18 to 34 years (*M* = 22.36, SD = 3.19 years). Advertisements requested participants for a study of the potential benefits of extreme music listening. It specified that participants should enjoy one or more extreme genres of music, such as heavy metal, punk, hardcore, and screamo, and listen to these at least 50% of the time they chose to listen to music. When individuals confirmed their participation, they were asked to bring along their personal music listening device to the laboratory. Three quarters of the participants (74%) were born in Australia, with the remainder born in New Zealand, USA, New Caledonia, South Africa, Indonesia, Sweden, and Oman. Seven participants were recruited via the online recruitment site (SONA) at the University of Queensland, receiving course credit for respective first year psychology courses. The remaining participants were recruited from the wider community via word of mouth and advertising on social media and community websites. They received a $10 iTunes voucher as compensation for their time and interest.

In regards to musical involvement, 41% of the participants currently played a musical instrument or sang, 51% attended live concerts on a regular basis (at least once a month), 44% composed music, and 23% had taught music, although it was not the same subsample engaging in all of these musical activities. Of the six activities included in the questionnaire, participants engaged in an average of three, which is similar to other research conducted in unselected adult samples (*authors, unpublished research*). The average number of years playing an instrument or singing was 6.19 years (SD = 5.22 years). The most commonly reported musical preferences were: classic metal 60%, death metal 17.5%, progressive metal 15%, punk 12.5%, power metal 7.5%, melodic metal 7.5%, folk metal 5%, black metal 5%, thrash metal 5%, death core 5%, and hard core 5%. Note that, as most participants indicated more than one preferred genre, the overall figure is above 100%. Table 1 shows means and SDs on the demographic, musical, and mood variables for the two conditions (music listening and control), and *t*-tests indicated no differences between the two conditions on these variables.

**Table 1:** **Sample characteristics of participants in the music and the control conditions**.

Variable	Music condition		Control condition		*t* value
	Mean	SD	Mean	SD	
Age in years	21.74	2.60	22.95	3.63	hbox1.193, ns
Years of singing/playing music	6.53	5.67	5.88	4.87	−0.385, ns
DASS_Depression	9.95	6.36	9.60	9.50	−0.133, ns
DASS_Anxiety	5.28	3.20	5.30	5.58	0.017, ns
DASS_Stress	12.00	6.25	11.59	7.73	−0.180, ns

### Procedure

Participants were randomly assigned to either the music or control condition before the study began. To avoid extraneous influences on heart rate, participants were asked to refrain from smoking, exercise, and drinking caffeinated and alcoholic beverages for at least 3 h before participating (this was checked with questions in the questionnaire). For the baseline heart rate recording, participants were given a diagram and instructions on how to attach their recording electrodes, and then asked to sit silently for 5 min and “not to think about anything in particular.” Following this, participants were asked to complete the first set of Positive and Negative Affect Scale (see PANAS in Measures) questions (T1). The experimenter then conducted the 16-min anger interview. Following this, participants completed the second set of PANAS questions (T2). Those assigned to the music condition were instructed to select song(s) of their preference from their personal music device, and were instructed to listen for 10 min. Although all participants were asked to bring their music devices to the experiment, this was the first moment that participants were told they would be listening to music. This was done to ensure that participants would select songs that they would typically listen to when feeling angry. Participants in the control condition were asked to “wait quietly for the next part of the experiment” and sat in silence for the next 10 min. All participants then completed the PANAS items for a third time (T3) followed by a structured interview about the emotional influence of music and the final questionnaires, which included the emotional influence of music questions, DASS, and demographic and musical involvement questionnaire (refer to measures). Participants were then debriefed. The average time for experiment completion was 50 min. Ethical clearance for the procedures and materials was granted through the university ethics committee.

### Measures

#### Demographics and Musical Involvement

Participants responded to demographic questions such as age and gender. Participants’ musical background and current musical involvement was assessed in a questionnaire consisting of seven dichotomous (yes/no) questions, such as “do you attend concerts or live music on a regular basis (i.e., at least once a month)?” Participants were also asked to identify the number of years (to the nearest 6 months) that they had played an instrument or sung during their lifetime. These seven items have been used in previous research by the authors and colleagues (*blinded for review*), and found to have good internal consistency (Cronbach’s α = 0.76).

#### Physiological Measure of Emotion

Heart rate was recorded according to published guidelines (Bernison et al., 2007): 10-mm pre-gelled Ag/AgCl disposable electrodes were attached over the lower rib on the left side of the torso and to the participant’s chest on both the right and left to record a lead III electrocardiogram (ECG). These leads were attached to a MP150 Biopac ECG system. Signals were digitized at 1000 Hz and saved for offline analyses. Heart rate, expressed as the number of beats per min (bpm), was sampled across the following time periods: 5 min baseline, 12 min anger induction, 10 min music or no music listening, and final 2 min of music listening and silence to yield an average heart rate (bpm) for each segment.

#### Modified Positive and Negative Affect Scale

Repeated measures of participants’ subjective emotional state was assessed with a modified version of the PANAS (Watson et al., 1988) using the time instructions “at the moment”. Participants were instructed to indicate how they felt at that very moment and rate 10 emotional words on a 5-point Likert scale from 1 (very slightly or not at all) to 5 (extremely). Five emotions had positive valence (e.g., “inspired” and “enthusiastic”), and five emotions had negative valence (e.g., “irritable,” “hostile,” and “guilty”).

#### Emotional Influence of Music

Participants were asked nine dichotomous (yes/no) questions during a structured interview, regarding the extent to which they listened to extreme music in order to change an emotion (e.g., “*when you are sad, do you listen to music that improves your mood?*”) or to fully experience an emotion (e.g., *“when you are angry, do you listen to music to fully experience that anger?”)*. The emotions were: happy, sad, angry, and anxious, with two extra items relating to “in love” and “well-being”. This questionnaire was adapted from a Likert-type scale version used in an international survey of 394 adults (*authors blinded for review*), which found an adequate internal consistency of items on the Change Emotions subscale of α = 0.73 and on the Experience Emotions subscale of α = 0.71 (Papinczak et al., 2015).

#### Depression, Anxiety, and Stress Scale

The Depression, Anxiety, and Stress Scale (DASS) is a 42-item questionnaire assessing symptoms of depression, anxiety, and stress over the past week (Lovibond and Lovibond, 1995). Questions were measured on a 4-point Likert-type scale from 0 (did not apply to me at all) to 3 (applied to me very much, or most of the time), with seven items summed to produce each of the three subscales scores: depression, anxiety, and stress. In our sample, the internal consistency values were: depression (α = 0.91), anxiety (α = 0.84), and stress (α = 0.90). Assessment of the DASS (42) on a non-clinical sample (*N* = 1771) (Crawford and Henry, 2003) found means for depression, anxiety, and stress to be 5.55 (SD = 7.48), 3.56 (SD = 5.39), and 9.27 (SD = 8.04), respectively.

#### Anger Interview

The stress interview proposed by Dimsdale et al. (1988), and modified by Lobbestael et al. (2008), was used for anger induction. The interview involved participants describing one or more events that produced a strong feeling of anger over a period of 16 min. Participants were presented with a list of topics to help with prompting their recall of angering scenarios, based on those used by Dimsdale et al. (1988) such as “partner/spouse”, “work/work colleagues,” and “finances”.. Other researchers, such as Burns et al. (2003) and Malatesta-Magai et al. (1992), have demonstrated the effectiveness of this technique in their respective studies, finding effects with only a 10-min interview.

#### Music and Headphones

As mentioned, participants were asked to bring in their personal music players to the laboratory, and those in the music listening condition were asked to play music from their own collection during the listening phase. Those in the experimental condition listening to their preferred music were provided with Sennheiser HD201 closed headphones.

## Results

### Self-report results

The means and SDs on the DASS (Table 1) show that symptoms of depression, anxiety, or stress were in the normal range, and there were no differences between participants in the two conditions. Responses to the nine questions about extreme music influence on emotions are displayed in Table 2. A majority agreed with the statements that they listened to extreme music to fully experience anger (79%) and to calm themselves down when feeling angry (69%). They also listened to extreme music to improve other negative moods such as sadness (74%) and less commonly, anxiety (33%). An overwhelming majority stated that they listen to extreme music to enhance their happiness (87%) and to enhance their well-being (100%).

**Table 2:** **Proportion (%) of participants responding “yes” to the music and emotional influence items**.

Question	Proportion (%)
Do you listen to music to enhance your happiness?	87.2
Do you think that listening to music enhances your wellbeing?	100.0
When you are sad, do you listen to music to fully experience your sadness?	51.0
When you are sad, do you listen to music that improves your mood?	74.0
When you are angry, do you listen to music to fully experience that anger?	79.0
When you are angry, do you listen to music to calm yourself down?	69.0
When you are anxious, do you listen to music to fully experience your anxiety?	0.0
Do you listen to music to calm yourself down when you are anxious?	33.0
When you are in love, do you listen to music to immerse yourself in those feelings?	67.0

### Experimental results

Means and SD on all of the emotion measures for participants in the two conditions are displayed in Table 3.

**Table 3:** **Means and SDs for heart rate and PANAS ratings**.

	Silence			Music		
	Baseline	Anger	Silence	Baseline	Anger	Music
Heart rate	81.45 (20.49)	87.51 (22.74)	77.67 (16.98)	77.39 (12.33)	94.13 (22.88)	89.66 (30.76)
Hostile	1.20 (0.70)	2.25 (1.07)	1.15 (0.37)	1.26 (0.73)	2.26 (0.99)	1.42 (0.61)
Irritable	1.15 (0.37)	2.10 (1.12)	1.30 (0.47)	1.42 (0.90)	2.26 (1.15)	1.16 (0.38)
Stressed	1.65 (0.75)	2.65 (1.04)	1.70 (0.92)	1.95 (1.03)	2.42 (0.90)	1.53 (0.70)
Relaxed	3.75 (0.91)	2.60 (0.94)	3.60 (0.82)	3.42 (0.96)	2.37 (0.68)	3.32 (0.82)
Active	1.95 (0.83)	3.00 (1.12)	1.90 (0.91)	2.00 (0.88)	2.89 (0.66)	2.84 (0.77)
Inspired	2.55 (0.95)	2.65 (1.09)	2.50 (1.05)	2.47 (0.77)	2.23 (0.92)	3.42 (0.96)

### Heart rate analyses

**Figure 1:**
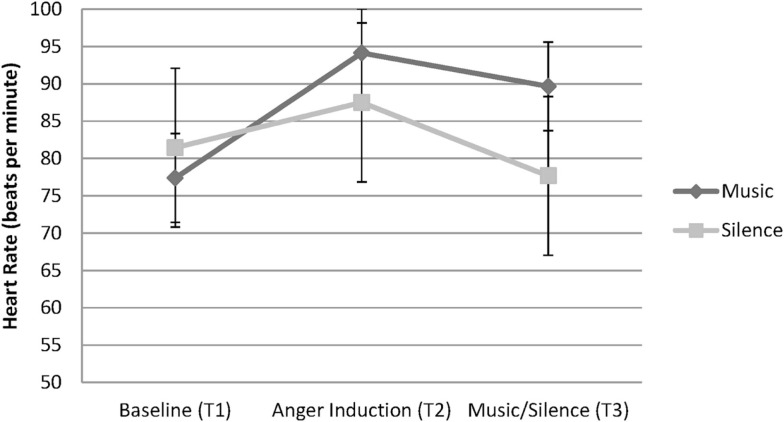
**Mean heart rate over the three time intervals for participants in the Music and Silence conditions**.

A 2 (Condition: Music vs. Silence) × 3 (Time: baseline, after anger induction, after music listening/silence) mixed repeated measures ANOVA was conducted to assess changes in heart rate during experimental periods, refer to Figure 1. A significant main effect of time was revealed, *F* (2, 74) = 8.54, *p* < 0.001, $\eta_{\text{p}}^{2}=0.\text{18.}$ There was no main effect of Condition; however, a significant Condition × Time interaction was found, *F* (2, 74) = 6.36, *p* = 0.003, $\eta_{\text{p}}^{2}=0.\text{15.}$ Tests of simple effects at each Condition revealed a significant effect of silence, *F* (2, 18) = 11.02, *p* = 0.001, $\eta_{\text{p}}^{2}=0.\text{55.}$ Simple comparisons revealed no significant difference between Time 1 and Time 2, or between Time 1 and Time 3. However, heart rate at Time 2 was significantly higher than at Time 3 (*p* = 0.001). A significant simple effect of time within the Music condition was also observed, *F* (2, 17) = 5.73, *p* = 0.013, $\eta_{\text{p}}^{2}=0.40.$ A simple comparison found significant differences of heart rate between Time 1 and Time 2, *p* = 0.008, where heart rate increased during the anger induction. Surprisingly, no significant difference was found between Time 1 and Time 3, *p* = 0.068. However, there was no significant difference among music listeners between heart rate during Time 2 and Time 3, *p* > 0.999, indicating that the increased heart rate following the anger induction was sustained for the music listeners, but not for those in the silence condition.

#### Subjective Ratings

A series of 2 (Condition: Music vs. Silence) × 3 (Time) mixed ANOVAs were conducted to compare PANAS self-reported emotions with Condition as the between-subjects factor. Self-reported ratings relevant to anger (PANAS hostile, irritable, and stress) were analyzed. In accounting for discriminant changes, the positively valenced emotions relaxed, active, and inspired were also analyzed. Where sphericity assumptions were not met, tests for Greenhouse-Geisser were reported. Means and SDs for self-reported emotions at each time point for each condition are shown in Table 3.

##### Hostile

**Figure 2:**
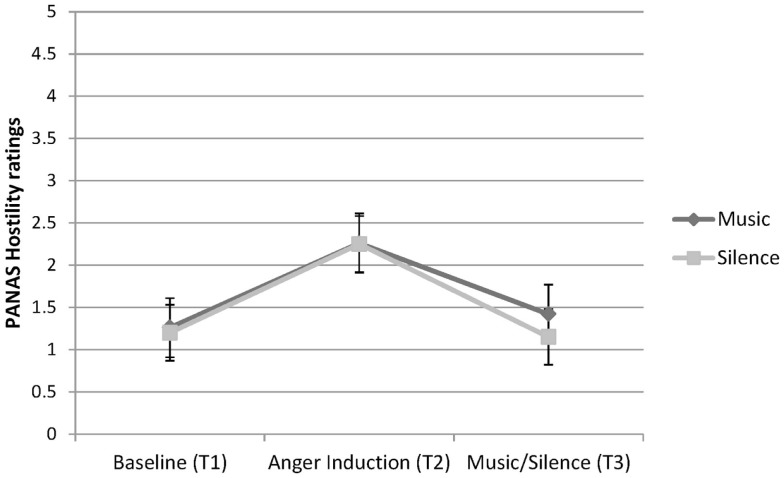
**PANAS hostility ratings at three time-points for participants in the Music and Silence conditions**.

A significant main effect was found for Time, *F* (1.50, 55.63) = 27.48, *p* < 0.001, $\eta_{\text{p}}^{2}=0.43.$ There was no main effect for Condition or a Condition × Time interaction. Pairwise comparisons of time points revealed no significant difference between Time 1 and Time 3; however, significant differences were observed between Time 1 and Time 2 (*p* < 0.001), and between Time 2 and Time 3 (*p* < 0.001). The greater ratings of hostility at Time 2, compared to Time 1 and Time 3, indicated that the anger induction worked, and that both music listening and silence resulted in decreased hostility. However, music listening was no different to silence, see Figure 2.

##### Irritable

A similar pattern of results emerged for PANAS irritable ratings. A significant main effect was revealed for time, *F* (1.69, 62.45) = 22.62, *p* < 0.001, $\eta_{\text{p}}^{2}=0.38$, with no significant main effect for Condition, or a Condition × Time interaction found. Pairwise comparisons of Time found no difference between Time 1 and Time 3; however, there were differences between Time 1 and Time 2 (*p* < 0.001), and between Time 2 and Time 3 (*p* < 0.001), such that greater ratings of irritability were observed at Time 2 compared to Time 1 and Time 3.

##### Stress

Baseline ratings of PANAS stress were higher than those for hostile and irritable, although the pattern of changes across time was consistent for the three PANAS emotions. A significant main effect of Time was found, *F* (1.69, 62.61) = 28.98, *p* < 0.001, $\eta_{\text{p}}^{2}=0.\text{54,}$ with no main effect of Condition or a Condition × Time interaction. Pairwise comparisons of Time found no difference between Time 1 and Time 3; however, the difference between Time 1 and Time 2 was significant (*p* < 0.001), as was the difference between Time 2 and Time 3 (*p* < 0.001), with greater ratings of stress at Time 2 compared to Time 1 and Time 3.

##### Relaxed

**Figure 3:**
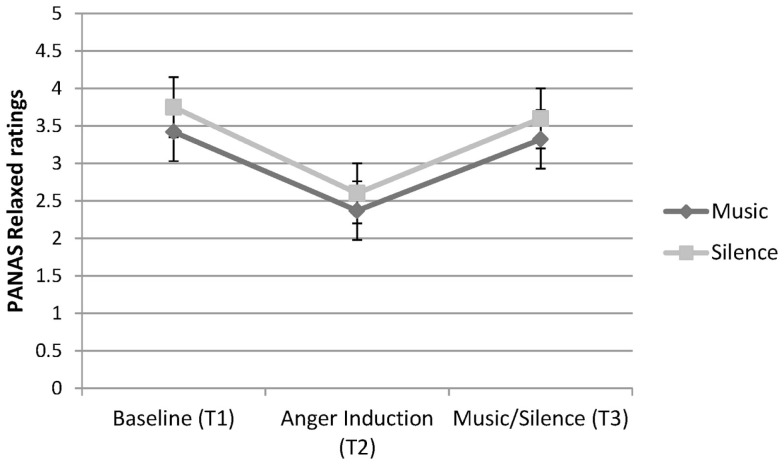
**PANAS ratings of relaxation at three time-points for participants in the Music and Silence conditions**.

An inverse pattern of results was found for the PANAS relaxed ratings, see Figure 3. No main effect was observed for Condition, or a Condition × Time interaction. A significant main effect of Time, however, was found, *F* (2, 74) = 22.62, *p* < 0.001, $\eta_{\text{p}}^{2}=0.38.$ Pairwise comparisons for Time found no significant difference between Time 1 and Time 3. However, there were differences observed between Time 1 and Time 2 (*p* < 0.001), and between Time 2 and Time 3 (*p* < 0.001), with participants reporting less relaxation at Time 2 compared to Time 1 and Time 3.

##### Active

A significant main effect was revealed for Time, *F* (2, 74) = 20, *p* < 0.001, $\eta_{\text{p}}^{2}=0.36,$ modified by a Condition × Time interaction, *F* (2, 74) = 6.98, *p* = 0.002, $\eta_{\text{p}}^{2}=0.16.$ No main effect for Condition was found. Tests of the simple effects of Time at each Condition were also conducted. The Silence group displayed significant simple effects of Time, *F* (2, 38) = 15.25, *p* < 0.001, $\eta_{\text{p}}^{2}=0.45$, and this was located between Time 1 and Time 2 (*p* = 0.002), and between Time 2 and Time 3 (*p* < 0.001), with participants feeling more active at Time 2 compared to Time 1 and Time 3 in the Silence condition. In the Music condition, the simple effects for Time was also significant, *F* (2, 36) = 12.54, *p* = < 0.001, η^2^ = 0.41. The key differences were found between Time 1 and Time 2 (*p* = 0.003), and between Time 1 and Time 3 (*p* < 0.001), with music listeners feeling more active after the anger induction and remaining active after music listening.

##### Inspired

**Figure 4:**
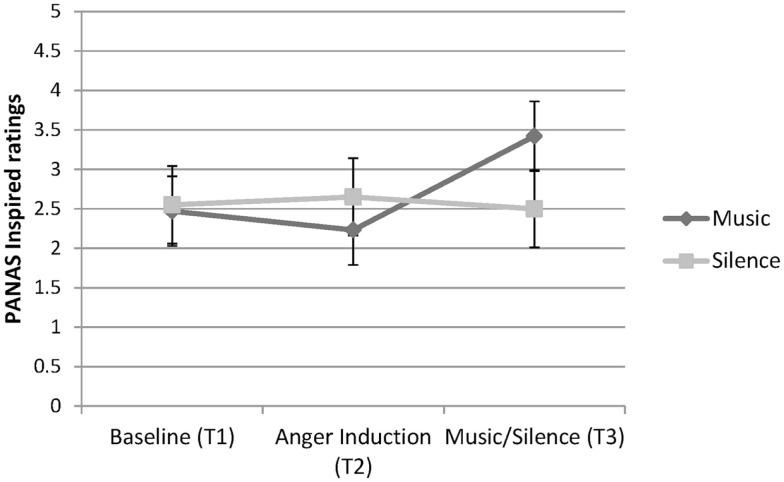
**PANAS inspired ratings for the participants in the Music and Silence conditions, showing a Time × Condition interaction**.

A significant main effect of Time was revealed, *F* (2, 74) = 4.74, *p* = 0.012, $\eta_{\text{p}}^{2}=0.11,$ as well as a significant Condition × Time interaction, *F* (2, 74) = 7.22, *p* = 0.001, $\eta_{\text{p}}^{2}=0.16.$ No main effect was found for Condition. A pairwise comparison for Time found no significant difference between Time 1 and Time 2, or between Time 1 and Time 3. However, inspiration ratings were greater at Time 3 compared to Time 2, *p* = 0.022, see Figure 4. The simple effects of Time at each Condition revealed no effects for Silence. The music group, however, displayed a significant effect of Time, *F* (2, 36) = 10.71, *p* < 0.001, η^2^ = 0.37. A simple comparison found no significant difference between Time 1 and Time 2. However, significant differences were observed between Time 1 and Time 3 (*p* = 0.021), and between Time 2 and Time 3 (*p* = 0.002), indicating that participants felt inspired after listening to their music.

#### Analysis of Music Selections

An analysis of the 46 pieces of music, the participants chose to listen to when angry, is displayed in Table 4. One song was removed because it could not be found; it was assumed that song title and/or artist were incorrectly recorded. Of the accurate song titles provided, 100% of music chosen was classified as “extreme” genre. Of the songs containing lyrics, only 50% contained aggressive themes or conveyed lyrics relating to anger, with the remaining songs containing lyrical themes including, but not limited to, isolation and depression. The tempo of the selected songs ranged from 80 to 181 beats per min, with over half (61%) of selections >100 bpm, representing high tempo music expected to have an energizing or arousing effect on the listeners. In all, less than a third of musical selections (28%) were both high arousal (>100 bpm) and contained themes of anger or aggression.

**Table 4:** **Analysis of the music participants played when angry**.

Song title	Artist	Genre	BPM	Violent/angry themes
1. Dawn of battle	Manowar	Heavy metal/epic power metal	100	Yes
2. The epic rage of furious thunder	Gloryhammer	Power metal	90	Yes^a^
3. Dark days	Parkway Drive	Metalcore	125	No
4. Dream run	Parkway Drive	Metalcore	88	No
5. 100 ways to hate	Five Finger Death Punch	Heavy metal	95	Yes
6. Opium of the people	Slipknot	Heavy metal	135	No
7. Adrenalize	In this moment	Heavy metal	132	No
8. Comanche	In this moment	Heavy metal	144	Yes
9. Beast within	In this moment	Heavy metal	90	Yes
10. Violence	A day to remember	Metalcore/pop punk		Yes
11. Live fast die beautiful	Escape the fate	Post-Hardcore	130	No
12. Forget about me	Escape the fate	Post-hardcore	95	No
13. Master of puppets	Metallica	Heavy metal	181	Yes
14. Vipers snakes and actors	Norma Jean	Metalcore	141	No
15. Night of the long knives	Marduk	Black metal	112	Yes
16. Judas Rising	Judas Priest	Heavy metal	134	Yes
17. Take no prisoners	Megadeth	Thrash metal	160	No
18. Mother	Danzig	Heavy metal	160	Yes
19. I am loco	Ill Nino	Heavy metal	87	No
20. Pull harder on the strings of your martyr	Trivium	Heavy metal	145	Yes
21. War of the gods	Amon Amarth	Melodic death metal	95	Yes
22. Roses on white lace	Arsis	Melodic death metal	160	No
23. Windrider	Ensiferum	Folk metal	93	Yes
24. Jumpdafuckup	Soulfly	Heavy Metal	152	Yes
25. Battle ready	Otep	Nu metal	107	Yes
26. The violation	Fleshgod Apocalypse	Death Metal	135	Yes
27. The egoism	Fleshgod Apocalypse	Death metal	150	Yes
28. My life for hire	A day to remember	Metalcore/pop punk	145	No
29. Not the american average	Asking Alexandria	Metalcore	140	No
30. What comes around	Ill Nino	Heavy metal	113	No
31. Corpse under glass	Morpheus Descends	Death metal	86	No
32. Suffer the gestalt	Mütiilation	Black metal	119	No lyrics
33. Attack	System of a down	Alternative metal	80	Yes
34. Kill rock n roll	System of a down	Alternative metal	92	Yes
35. Sugar	System of a down	Alternative metal	131	Yes
36. It snows in hell	Lordi	Heavy metal	92	No
37. Astral path to supreme majesties	Inquisition	Thrash/black metal	92	No
38. Command of the dark crown	Inquisition	Thrash/black metal	95	Yes
39. Cosmic invocation rites	Inquisition	Thrash/black metal	116	No
40. Pravus	Meshuggah	Extreme metal	133	Yes
41. Obzen	Meshuggah	Extreme metal	85	No
42. Asylum	Disturbed	Heavy metal	180	No
43. Killing in the name	Rage against the machine	Rap metal	93	Yes
44. Dragula	Rob Zombie	Heavy metal/industrial metal	125	No
45. Of matter-retrospect	Tesseract	Progressive metal	90	No
46. Of energy-singularity	Tesseract	Progressive metal	108	No

*^a^Lyrical themes of anger/aggression, but contextually not aggressive*.

## Discussion

### Extreme music and anger

The purpose of this research was to test two alternative sets of hypotheses regarding the relationship between extreme music and anger under controlled experimental conditions. The first set of hypotheses followed an “extreme music causes anger” line of reasoning, and the second set of hypotheses followed an “extreme music matches and helps to process anger” line of reasoning. The results overall were supportive of the latter. Among our sample of extreme music fans in the normal range on symptoms of depression, anxiety, and stress, the majority reported that they listened to extreme music for a range of emotional effects – most pertinently to fully experience anger and to calm themselves down when feeling angry.

These reports were supported by the experimental results. The anger induction was successful, as shown in increased ratings of hostility and irritability and increased heart rate at the end of the anger interview. Those who listened to music when angry did not show an increase in heart rate or subjective hostility and irritability. Rather, they showed a decrease in subjective hostility and irritability that was equivalent to those who sat in silence. Heart rate stabilized but did not continue to rise, suggesting that the music that participants selected when angry matched their physiological arousal and allowed them to fully experience it. In the silence condition, heart rate reduced after the anger interview, returning to baseline. These findings are consistent with Gowensmith and Bloom (1997) finding that heavy metal music was highly arousing to both fans and non-fans but did not cause an increase in subjective anger in fans. The findings are counter to the claims that extreme music causes anger and promotes aggressive behavior (Stack et al., 1994; Arnett, 1996).

In addition, the results showed that listening to metal music relaxed participants as effectively as sitting in silence. Ratings of relaxation decreased during the anger induction but increased again during music listening or silence. This result expands on earlier research by Labbé et al. (2007) who reported that personally selected music of any genre is just as relaxing as (experimenter selected) classical music. Unfortunately, because a similar relaxation response was found in both conditions, it is unclear whether it was the music or simply the passage of time after the anger induction that may have increased feelings of relaxation. Nevertheless, ratings on two other positive emotions, active and inspired, further demonstrate that music listening helped participants to feel these positively valenced emotions. Active feelings increased in all participants during the anger induction, consistent with the idea that anger activates approach motivation (Carver and Harmon-Jones, 2009). Active feelings then decreased for participants in the silence condition; yet, they continued to increase in the music listeners. Ratings of feeling inspired were relatively flat from baseline to anger induction for both conditions and were unchanged for those who sat in silence. In contrast, participants who listened to their selected extreme music experienced a significant increase in feelings of inspiration. These effects of extreme music on increasing physiological arousal and subjective inspiration are echoed in other research showing that music can evoke the experience of power – an effect that appears to be independent of musical genre and whether or not the music contains lyrics (Hsu et al., 2015). Taken together, the findings support the view that extreme music listeners use music to regulate their anger and to feel active and inspired. This emotion regulation effect is similar to that found in some research on sad music listening (Saarikallio and Erkkila, 2007; Vuoskoski et al., 2012). For instance, Van den Tol and Edwards (2013) found that people often engaged in sad music listening when sad in order to fully experience their negative affect and to enhance their mood. Indeed, participants in our study also reported listening to extreme music to improve their mood when feeling sad.

### What did angry participants listen to?

A secondary aim for the study was to analyze what participants in the music condition selected from their own playlists to listen to when they were angry. It was predicted that angry participants would select extreme music from their playlists that matched their anger in terms of high tempo and angry lyrics. The analysis confirmed that all participants chose to listen to extreme music after the anger induction. The tempo and lyric findings were interesting in that half of the chosen songs contained lyrical themes of anger or aggression, with the remainder contained other themes including, but not limited to, isolation and sadness. It is difficult to account for this finding without knowing the detailed content of the angry memories that participants evoked during the anger interview. It is possible that their memories incorporated complicated feelings including anger and sadness and that their selected music matched those feelings. It is also possible that many participants did not select music on the basis of the lyrics – rather on the basis of the instrumental sounds or other musical characteristics. In terms of tempo, the chosen songs had a range of tempo with only 61% having a tempo that would be considered highly arousing (100 beats per min or over). Furthermore, less than a third of all songs possessed both angry themes and high arousal tempo. Potentially, other mechanisms may have linked the music with participants’ emotional response, such as episodic memory, emotional contagion, or a brain stem response to the acoustic characteristics of the music (Juslin and Västfjäll, 2008; Juslin et al., 2010).

Unfortunately, it was not possible to conduct an analysis directly linking participants’ heart rate to the songs they listened to because we wanted participants to engage in naturalistic music listening and they listened to multiple songs (with varying tempos) for various lengths of time during the 10 min period. We did not have markers on the heart rate recording of which songs were listened to for which periods, and therefore the only analysis available was a summary analysis of the music they listened to (unlinked to their heart rates). Further research is required to explore whether there is a direct relationship between song tempo and heart rate among angry extreme music fans, as has been found in other samples (e.g., Etzel et al., 2006).

Extreme music fans reported using their music to enhance their happiness, to immerse themselves in feelings of love, and agreed that their music enhanced their well-being. What each of these responses indicates is that extreme music listeners appear to be using their music listening for positive self-regulatory purposes. Although this effect cannot be generalized to non-fans, it nevertheless lends support to a growing body of research about everyday music listening and emotion regulation (Saarikallio, 2011; Thoma et al., 2012; Papinczak et al., 2015).

### Practical implications

Given that some correlational studies have reported an association between extreme music and anger, aggression and delinquency, it is understandable that some parents, teachers, and health practitioners have been concerned about their clients or students listening to extreme music and what this might mean. Earlier studies showed that an individual’s music preference is capable of biasing clinical judgment – for example, Rosenbaum and Prinsky (1991) contacted clinicians at 12 psychiatric hospitals posing as a concerned parent of a (fictitious) adolescent male who listened to heavy metal but they made no mention of symptoms of any mental illness. Ten of the services (83%) recommended admitting the adolescent to hospital. The results of our study indicate that responses like these are unjustified. On the contrary, the results show that extreme music may be used to recover from anger and to enhance emotional and mental health.

Practically, this research has various uses in applied settings. For example, greater understanding of anger processing through music may be beneficial within schools. Young people, in particular adolescents, are the greatest consumers of music (North and Hargreaves, 1999; North et al., 2000). Thus, allowing students who are angry and upset to listen to their preferred music (including extreme genres) for 10 min may assist in self-regulation of these moods and result in increased positive affect. Moreover, these findings are extremely useful in clinical settings. Music-based interventions have been found to be effective in the treatment of a range of disorders that commonly involve emotional volatility including the psychoses (Gold et al., 2009), post-traumatic stress disorder (Zoteyva et al., 2015), and substance misuse (Baker et al., 2012; Short and Dingle, 2015). The use of extreme music in therapy may also result in increased engagement and participation in therapy for fans of these genres (Dingle et al., 2008).

### Limitations and future directions

Although these results showed that extreme music matches and helps to regulate anger – this effect may be particular to fans of extreme music that are not experiencing any symptoms of distress. Further research is required to examine whether the findings generalize to fans experiencing psychological or behavioral problems. It is also important to note that the study was carried out in a laboratory under controlled conditions and with only the participant and experimenter present. Further, as participants were recruited with an advertisement for the “potential benefits” of extreme music, it partially revealed the study aims possibly leading to bias. In light of the results, it would have been beneficial to have included a third condition in which participants listened to a non-problem music genre in order to control for the general arousing effects of listening to music of any kind.

It is unknown what might happen to participants’ emotions if they listened to extreme music for prolonged periods, or what their emotional and arousal levels were half an hour or more after listening had ceased. The study would need to be replicated and extended to include a fourth time point in order to clarify this question. It is not clear from these findings how a naturalistic setting (such as at a social gathering or concert) might influence the link between extreme music listening and anger processing. Further research adopting experience sampling methods might shed light on this (Juslin et al., 2008). Finally, we did not measure individual difference factors such as personality, tendency to ruminate, and other emotion regulation strategies in this study – factors that have been implicated in emotional responses to music in other research (Chin and Rickard, 2014; Garrido and Schubert, 2015). Such musical, contextual, and listener variables may all contribute in some way to listeners’ emotional responses, as has been found in previous research (Juslin and Sloboda, 2010).

What may be of interest for future research is how extreme music fans use music listening to process other emotions such as sadness and anxiety? Just over half of the participants in this study indicated that they listen to extreme music to fully experience sadness, and three quarters said they listen to improve their mood when feeling sad. However, there is currently a lack of research putting this to a direct test using experimental manipulation of sad mood. Only a third used music to calm down when anxious, which may reflect the highly arousing nature of the music. It would be interesting to find out if extreme music fans use other genres of music or other non-musical strategies (such as exercise or talking to someone) to regulate their anxiety (Thayer et al., 1994).

## Conclusion

This study found that extreme music fans listen to music when angry to match their anger, and to feel more active and inspired. They also listen to music to regulate sadness and to enhance positive emotions. The results refute the notion that extreme music causes anger but further research is required to replicate these findings in naturalistic social contexts, and to investigate the potential contributions of individual listener variables on this relationship between extreme music listening and anger processing.

## Conflict of Interest Statement

The authors declare that the research was conducted in the absence of any commercial or financial relationships that could be construed as a potential conflict of interest.
